# Each British Woman to Be Stirred up?

**Published:** 1918-11-23

**Authors:** Alice Petitt

**Affiliations:** Member of the College of Nursing. P.S.—I am not ashamed of my name; you may publish it. 183 Cheetham Hill Road, Manchester.


*To the Editor of* THE HOSPITAL.

### Each British Woman to be Stirred up?

*To the Editor of* THE HOSPITAL.

SIR,—You ask for answers to the question, "Is every woman a nurse?" No, certainly not. Every woman may have certain qualities that go for the making of a nurse, but if she has not the heart and mind of a nurse she will never make one. There may be power behind the machine, but unless the mechanism is there to do the particular work required the power is useless. So with the nurse. Need more be said? I have been curious to see what reply "Ierne" would make to the severe criticisms lately applied to him, or her. I am satisfied with the reply, and would like to add my opinion. Such weak-minded criticisms have only proved that nurses, some at least, be they matrons or sisters, have gone about with their house on their back like snails, leaving their trail, which is only a slimy track. They cannot see the difference between loyalty and deaf and dumb and blind loyalty, like the Germhuns'. See what such loyalty has brought them to! We have suffered from this sort of loyalty too long. By the way, I wonder how much longer the Bedford Fenwick loyalty will last! Thanks to the College of Nurses, our blindness is nearly cured, and before it is too late. One can see that healthy discontent is absolutely necessary in all cases of reform. What did the Suffragists do but make women discontented with the unfair conditions of control and employment? And the "vote" is the result, and even Bedford Fenwicks do not despise that. We have in the thin end of the wedge now, and women's position will not rattle any more. I would like to see this healthy discontent spread more rapidly even amongst the industrial housewife. To go so far as to make her discontented and disloyal, in the same way, to her husband and household. The treatment so often given to a wife of this class is unbearable. She is not the servant and slave of a lord and master. She is, or should be, the companion and helpmate of a man. His own choice and her assent, therefore a mutual contract. I admit that I am doing my best to set this sort of discontent on foot when I know it will be received with common-sense. Nurses sign a contract that they will give so many years of their life in return for a mere pittance, and a guarantee that they shall be taught a profession that will enable them to be at least a decent wage-earning citizen. Leaving out all sentiment, if hospitals have trained women unfit for this profession that is their look-out, not the nurses', as a man who makes a bad choice in a wife. But because we want to remedy this and pick out the tares from the wheat, certain people have got their back up and say "Wait, and the good will come out top," but it never has. If these people would remove the beam from their eye they would see better. I wonder what they are afraid of. A contract is an agreement between two parties or more as may be, and conditions are binding, "except for Germhuns." The conditions mean a trained person fitted to do certain work. Very well, let us have a universal condition, then there need be no grumbling, no disloyalty, no disparity of conditions.—I am, Sir, yours faithfully,

ALICE PETITT,
Member of the College of Nursing.

P.S.—I am not ashamed of my name; you may publish it.

183 Cheetham Hill Road, Manchester.

November 16, 1918.

